# B-Mode Ultrasound Radiomics for Differentiating Benign and Malignant Small Hyperechoic Renal Masses: An Exploratory Single-Center Experience

**DOI:** 10.3390/jimaging12070315

**Published:** 2026-07-10

**Authors:** Fabrizio Urraro, Nicoletta Giordano, Vittorio Patanè, Marco Piscopo, Giovanni Ciani, Giovanni Balestrucci, Maria Chiara Brunese, Anna Russo, Mario Sansone, Alfonso Reginelli

**Affiliations:** 1 Department of Life Sciences, Health and Health Professions, Link Campus University, 00165 Rome, Italy; f.urraro@unilink.it; 2 Precision Medicine Department, University of Campania “Luigi Vanvitelli”, 80138 Naples, Italy; vittorio.patane@unicampania.it (V.P.); dott.giovanniciani@gmail.com (G.C.); mariachiara.brunese@unicampania.it (M.C.B.); annarusso81@yahoo.it (A.R.); alfonso.reginelli@unicampania.it (A.R.); 3 Radiology Unit, Pineta Grande Hospital, 81030 Castel Volturno, Italy; mp.marcopiscopo@gmail.com (M.P.); giovanni.balestrucci@studenti.unimol.it (G.B.); 4 Department of Electrical Engineering and Information Technology, University of Naples “Federico II”, 80125 Naples, Italy; mario.sansone@unina.it

**Keywords:** ultrasound, radiomics, B-mode imaging, small renal mass, hyperechoic renal mass, renal cell carcinoma, angiomyolipoma

## Abstract

**Introduction**: Small hyperechoic renal masses are frequently detected incidentally on conventional ultrasound and are often presumed to represent benign lesions, particularly angiomyolipomas. However, malignant renal tumors, including renal cell carcinoma, may also appear hyperechoic when small, creating a diagnostic challenge at first-line imaging. This study aimed to evaluate the feasibility and exploratory diagnostic performance of B-mode ultrasound radiomics for differentiating benign and malignant small hyperechoic renal masses. **Methods**: This retrospective single-center study included adult patients with incidentally detected small hyperechoic renal masses measuring ≤3 cm and examined between July 2022 and April 2025. All lesions underwent standardized B-mode ultrasound assessment and multidisciplinary review. Final diagnosis was established by histopathology when available or by longitudinal ultrasound follow-up stability for lesions considered benign. Lesions were manually segmented on representative B-mode DICOM images, and original radiomic features were extracted using PyRadiomics version 3.0 according to standardized definitions compatible with the Image Biomarker Standardisation Initiative framework. A total of 114 original radiomic features were extracted from each lesion. The primary comparison was benign versus malignant lesions. Diagnostic performance was assessed using feature-level receiver operating characteristic analysis. **Results**: Forty-two lesions were included in the final radiomic cohort, including 26 malignant renal cell carcinomas and 16 benign angiomyolipomas. Malignant lesions included papillary renal cell carcinoma, chromophobe renal cell carcinoma, and clear-cell renal cell carcinoma. All malignant lesions were histologically confirmed. Among benign lesions, 14 angiomyolipomas were classified based on longitudinal ultrasound stability, whereas 2 were confirmed by ultrasound-guided percutaneous biopsy after mild dimensional increase during imaging surveillance. Among the extracted radiomic features, firstorder_Variance and firstorder_MeanAbsoluteDeviation showed the highest exploratory discriminatory performance, each achieving an area under the receiver operating characteristic curve of 0.837. Both features are first-order measures of gray-level dispersion within the segmented lesion. Higher values were observed in malignant lesions, suggesting greater intralesional grayscale heterogeneity compared with benign angiomyolipomas. **Conclusions**: B-mode ultrasound radiomics is feasible for the quantitative assessment of small hyperechoic renal masses and may provide complementary information for differentiating benign angiomyolipomas from malignant renal cell carcinomas. firstorder_Variance emerged as a representative candidate imaging biomarker of grayscale dispersion, with firstorder_MeanAbsoluteDeviation showing concordant performance as a related dispersion measure. These findings should be considered preliminary and hypothesis-generating and require validation in larger multicenter cohorts before clinical implementation.

## 1. Introduction

The widespread use of abdominal imaging has led to an increasing incidental detection of small renal masses, many of which are first identified during conventional ultrasound examinations performed for unrelated indications [1,2,3]. While simple renal cysts can usually be characterized by high confidence on grayscale ultrasound, solid small renal masses remain diagnostically challenging, particularly when they appear hyperechoic [4,5,6].

In daily radiological practice, a small well-defined hyperechoic renal lesion is often presumed to represent a benign angiomyolipoma [7,8,9,10,11,12]. Although this assumption is frequently correct, hyperechogenicity is not specific for benign disease. Malignant renal tumors, including renal cell carcinoma, may also appear hyperechoic, especially when small. Therefore, visual echogenicity alone cannot reliably distinguish benign from malignant small renal masses, and this diagnostic overlap may influence subsequent patient management, including surveillance, additional imaging, biopsy, ablative treatment, or surgery [13,14].

Contrast-enhanced CT and MRI remain central for the characterization and staging of indeterminate renal masses [15,16]. Nevertheless, conventional B-mode ultrasound continues to play an important first-line role because it is widely available, inexpensive, repeatable, and free of ionizing radiation [17,18]. Its main limitation is that interpretation remains partly qualitative, operator-dependent, and influenced by technical and patient-related factors. In this setting, quantitative tools capable of supporting early risk stratification at the first-line ultrasound stage may be clinically valuable [19,20].

Radiomics offers a potential approach by converting medical images into quantitative features that describe intensity distribution, morphology, and texture patterns [21,22,23,24]. These features may capture subtle differences in lesion composition and intralesional heterogeneity that are not consistently appreciable on visual assessment alone [25,26,27,28]. Although radiomics has been widely investigated in CT, MRI, and PET imaging, its application to ultrasound is less mature and methodologically more challenging because ultrasound images are strongly affected by acquisition parameters, transducer frequency, gain, depth, focus, acoustic window, operator technique, and vendor-specific post-processing [29,30,31,32,33,34,35,36,37]. For this reason, ultrasound radiomics requires careful standardization of image acquisition, image selection, segmentation, preprocessing, and feature extraction [38,39,40].

The originality of the present study lies in its focused clinical scenario. Rather than evaluating renal masses as a broad heterogeneous category, this study specifically addresses small hyperechoic renal masses, a subgroup in which conventional ultrasound is frequently the first imaging modality but visual assessment may be insufficient for confident benign versus malignant differentiation. We hypothesized that malignant small hyperechoic renal masses may show greater intralesional grayscale heterogeneity than benign lesions, even when their overall echogenic appearance is similar on visual assessment. Therefore, the aim of this exploratory single-center study was to evaluate the feasibility and preliminary diagnostic performance of radiomic features extracted from standardized B-mode ultrasound images for differentiating benign and malignant small hyperechoic renal masses, with the goal of identifying candidate imaging biomarkers for future validation studies.

## 2. Materials and Methods

This was a retrospective, single-center study conducted at the University Hospital “Luigi Vanvitelli”, Naples, Italy. The study protocol was approved by the local ethics committee of the University Hospital “Luigi Vanvitelli” (Naples, Italy; Prot. 158/i/2022). The requirement for written informed consent was waived by the ethics committee because of the retrospective nature of the study.

The study was designed as an exploratory radiomic feature-level analysis. Its aim was not to replace histopathology, cross-sectional imaging, or expert radiological interpretation, but to evaluate whether quantitative features extracted from conventional B-mode ultrasound images could provide additional information in the characterization of small hyperechoic renal masses, a setting in which qualitative visual assessment may be uncertain.

### 2.1. Patient Population

Patients examined between July 2022 and April 2025 were retrospectively reviewed. Eligible patients were adults with an incidentally detected small hyperechoic renal mass, defined as a solid hyperechoic renal lesion with a maximum diameter of 3 cm or less on conventional ultrasound.

Inclusion criteria were: Age ≥ 18 years; presence of a small hyperechoic renal mass measuring ≤3 cm on initial ultrasound; standardized ultrasound acquisition; adequate B-mode image quality for segmentation and radiomic analysis; availability of diagnostic confirmation by histopathology or longitudinal imaging follow-up when appropriate; and multidisciplinary case review.

Exclusion criteria were: Inadequate ultrasound image quality; incomplete imaging data; uncertain final diagnosis; severe comorbidities with life expectancy < 1 year; pregnancy; and absence of sufficient diagnostic confirmation.

The final cohort selection and diagnostic distribution are reported in the Results Section (Section 3).

### 2.2. Reference Standard and Diagnostic Groups

Final diagnosis was established by histopathology when available, including surgical resection or percutaneous renal mass biopsy. Histopathological confirmation was required for all lesions classified as malignant.

For lesions classified as benign, histopathology was used when available. In lesions not referred for biopsy or surgery because of benign imaging appearance and multidisciplinary assessment, longitudinal ultrasound stability was accepted as a surrogate reference standard. Benignity was defined by the absence of relevant dimensional increase or morphological change during imaging follow-up. Follow-up ultrasound was initially performed at 3 months and subsequently continued for at least 18 months when histopathological confirmation was not available.

For the primary analysis, lesions were classified into two diagnostic groups: benign and malignant. The malignant group included renal cell carcinoma subtypes, whereas the benign group included angiomyolipomas. This binary grouping was selected to address the clinically relevant question of whether B-mode ultrasound radiomic features could help differentiate malignant from benign small hyperechoic renal masses.

### 2.3. Ultrasound Acquisition

All ultrasound examinations were performed using the same ultrasound system, a Canon Aplio a550 platform (Canon Medical Systems, Bovenkerkerweg 59, 1185XB Amstelveen, The Netherlands), with a convex-array transducer operating at 1–6 MHz. Examinations were performed according to a standardized abdominal ultrasound protocol.

All examinations were performed using a standardized renal B-mode protocol based on the abdominal/renal preset of the ultrasound system. The convex-array transducer was operated within the available 1–6 MHz range, with a typical renal imaging frequency of approximately 3.5–5 MHz, selecting the highest frequency that provided adequate renal penetration. Image depth was standardized to include the entire kidney, the target lesion, and adjacent renal parenchyma, while avoiding unnecessary excess depth; in most cases, depth ranged between 12 and 16 cm, with limited adjustment according to patient habitus and renal position. The focal zone was placed at the level of the lesion or immediately below it.

Overall gain and time-gain compensation were maintained according to the renal preset whenever possible and were adjusted only minimally to obtain homogeneous visualization of the renal parenchyma and complete depiction of the hyperechoic lesion, while avoiding under-gaining, signal saturation, or clipping of intralesional grayscale information. Image optimization parameters, including tissue harmonic imaging, speckle reduction, dynamic range, persistence, and compounding settings, were kept according to the standardized renal preset of the ultrasound platform across the included examinations. No image adjustments were performed for radiomic purposes, and all images were acquired before radiomic feature extraction.

### 2.4. Image Selection and Segmentation

Ultrasound images were exported from the institutional PACS in DICOM format and transferred to an offline workstation. For each lesion, the most representative B-mode image was selected by a radiologist with more than 10 years of experience in abdominal ultrasound, blinded to the final diagnosis.

The most representative image was defined as the B-mode frame that provided the best visualization of the lesion in its predominant dimension. Selection criteria included complete lesion depiction, optimal relative and absolute contrast between the lesion and adjacent renal parenchyma, adequate definition of lesion margins, absence of major acoustic artifacts, and suitability for manual segmentation.

Images were imported into ITK-SNAP version 3.8.0 for manual segmentation [41]. Segmentation was performed by one experienced radiologist by delineating the entire visible lesion on the selected B-mode image. Adjacent renal parenchyma, extralesional structures, acoustic artifacts, and shadowing unrelated to the lesion were excluded from the region of interest. The region of interest was drawn to include the full visible lesion area rather than a selected internal portion, in order to capture global intralesional heterogeneity.

Manual segmentation was selected because small renal lesions on ultrasound may show heterogeneous echogenicity, partially indistinct margins, posterior artifacts, and variable conspicuity relative to adjacent renal parenchyma. In this setting, automatic or semi-automatic segmentation may be unreliable. Manual segmentation, although time-consuming and operator-dependent, allows careful anatomical control of the region of interest, which is particularly important for small lesions.

**Figure 1:**
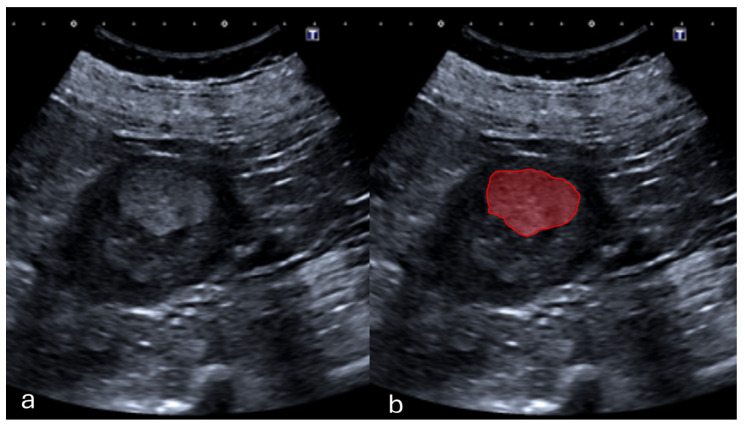
Representative manual segmentation example of a small hyperechoic renal mass. (**a**) B-mode ultrasound image showing a small hyperechoic mesorenal renal lesion. (**b**) Corresponding manual segmentation with the region of interest overlaid on the lesion. The ROI was drawn to include the visible lesion area while excluding adjacent renal parenchyma and extralesional structures. Histopathological analysis confirmed clear-cell renal cell carcinoma. This example illustrates the manual segmentation approach used for radiomic feature extraction and highlights the intralesional grayscale heterogeneity assessed by first-order radiomic features.

The segmentation workflow was applied consistently across all cases. Whenever lesion margins were ambiguous, segmentation prioritized the visible lesion boundary and excluded adjacent renal parenchyma to avoid contamination of intensity-based features. A representative segmentation example is provided in Figure 1, showing a small hyperechoic mesorenal renal mass on B-mode ultrasound before and after manual ROI delineation.

The lesion was histologically confirmed as clear-cell renal cell carcinoma. This example illustrates how the region of interest was drawn to encompass the visible hyperechoic lesion while excluding adjacent renal parenchyma, acoustic artifacts, and extralesional structures.

### 2.5. Radiomics Feature Extraction

Radiomic feature extraction was performed using PyRadiomics version 3.0, according to standardized feature definitions compatible with the Image Biomarker Standardisation Initiative framework [42,43].

For each lesion, the manually segmented B-mode region of interest was used as input for radiomic analysis. Images were analyzed as two-dimensional grayscale ultrasound images, with explicit 2D feature extraction. Only original radiomic features were extracted; no filtered features, including wavelet, Laplacian of Gaussian, square, square-root, logarithmic, or exponential transformations, were included.

Images were analyzed as two-dimensional grayscale ultrasound images. No spatial resampling was performed, and images were analyzed at their native spatial resolution. Before feature extraction, intensity normalization was performed within the PyRadiomics pipeline by setting normalize = True for all cases. The same normalization setting was applied consistently to all lesions and diagnostic groups.

In this context, normalization refers to standardized post-export radiomic preprocessing of the grayscale image before feature extraction, rather than to manual adjustment of the ultrasound image. This step was used to reduce non-biological variability related to grayscale scaling and image export. Because the same preprocessing settings were applied to the entire cohort, first-order dispersion features, including firstorder_Variance and firstorder_MeanAbsoluteDeviation, were interpreted as measures of relative intralesional grayscale heterogeneity within a standardized preprocessing framework.

Gray-level discretization was performed using a fixed bin width approach, with a bin width of 25 gray levels, for matrix-based texture feature extraction. The same preprocessing and feature extraction settings were applied to all lesions, regardless of final diagnostic group. All preprocessing choices were fixed before statistical analysis.

Technical variability was minimized through standardized ultrasound acquisition, use of a single ultrasound platform, DICOM image export, blinded selection of representative B-mode frames, and application of identical radiomic extraction settings across the entire cohort.

**Figure 2:**
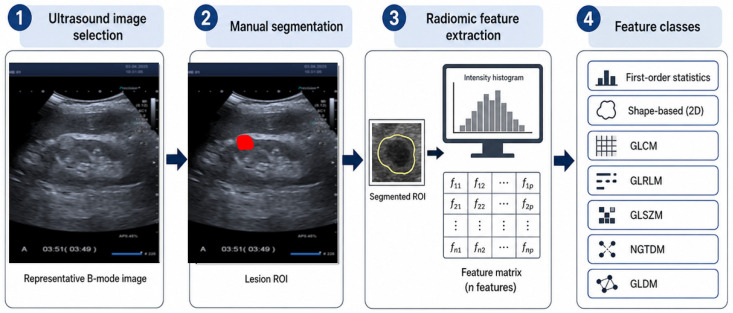
Radiomic workflow for B-mode ultrasound analysis of small hyperechoic renal masses. Representative B-mode images were selected, lesions were manually segmented to obtain a two-dimensional region of interest, and original radiomic features were extracted from the segmented lesion. Extracted feature classes included first-order statistics, two-dimensional shape-based features, gray-level co-occurrence matrix, gray-level run-length matrix, gray-level size-zone matrix, neighboring gray-tone difference matrix, and gray-level dependence matrix features.

A total of 114 original radiomic features were extracted from each lesion. Feature classes included first-order statistics, two-dimensional shape-based features, gray-level co-occurrence matrix features, gray-level run-length matrix features, gray-level size-zone matrix features, neighboring gray-tone difference matrix features, and gray-level dependence matrix features. The overall radiomic workflow adopted in the present study is summarized in Figure 2.

The extracted radiomic features were grouped into predefined classes reflecting complementary quantitative properties of the segmented lesion, including gray-level intensity distribution, two-dimensional morphology, and spatial texture organization, as summarized in Table 1.

For methodological clarity, firstorder_Variance and firstorder_MeanAbsoluteDeviation were interpreted as first-order intensity-distribution features rather than spatial texture features. Both quantify the dispersion of gray-level values within the segmented region of interest and therefore reflect global intralesional grayscale heterogeneity.

### 2.6. Statistical Analysis

Statistical analysis was performed as an exploratory feature-level analysis. The primary comparison was between benign and malignant small hyperechoic renal masses. Continuous radiomic variables were evaluated according to the final diagnostic group.

For each radiomic feature, discriminatory performance was assessed using receiver operating characteristic (ROC) analysis. The area under the ROC curve (AUC) was used as the primary performance metric. Features with the highest AUC values were considered candidate radiomic biomarkers, provided that they also showed biological interpretability and methodological plausibility.

Given the limited sample size and the preliminary single-center design, no multivariable predictive model was developed. Given the limited sample size and the preliminary single-center design, no multivariable predictive model was developed. Although multivariable machine-learning classifiers, such as support vector machines or random forests, may be useful in larger radiomic studies, their application in the present cohort would have been associated with a substantial risk of overfitting because of the small number of lesions relative to the number of extracted features. Therefore, the analysis was intentionally restricted to a feature-level exploratory framework. Continuous radiomic variables were not used to train a classifier; instead, each feature was evaluated by ROC analysis to identify interpretable candidate biomarkers with promising discriminatory performance. This conservative strategy was chosen to avoid overinterpretation of high-dimensional radiomic data in a small single-center cohort. The analysis therefore focused on identifying individual radiomic features with promising exploratory discriminatory performance.

For the best-performing features, 95% confidence intervals for AUC values were estimated using 2000 bootstrap resampling. Because 114 radiomic features were evaluated in a limited cohort, multiple-comparison control was performed using the Benjamini–Hochberg false discovery rate procedure. For each radiomic feature, exploratory univariate comparison between benign and malignant lesions was performed, and the resulting *p* values were adjusted across the full set of extracted features.

FDR-adjusted q values were used to assess whether feature-level findings remained robust after correction for multiple testing. Given the exploratory design of the study, the FDR analysis was not used to define a definitive diagnostic signature or a clinically applicable cut-off. Instead, it was used as a sensitivity analysis to avoid overinterpretation of feature-ranking results. Radiomic features were interpreted as candidate biomarkers only in the presence of favorable exploratory AUC, biological plausibility, methodological interpretability, and transparent reporting of multiple-comparison-adjusted results.

Unsupervised heat-map visualization and hierarchical clustering were used as descriptive tools to explore radiomic pattern distribution between benign and malignant lesions. These analyses were considered supportive and exploratory and were not used as standalone evidence of diagnostic accuracy.

## 3. Results

### 3.1. Patient and Lesion Characteristics

A total of 44 patients with incidentally detected small hyperechoic renal masses were initially screened. Two patients were excluded because the available B-mode images did not meet the quality or standardization requirements for reliable radiomic feature extraction. The final radiomic cohort therefore included 42 lesions.

Of these, 26 lesions were malignant renal cell carcinomas and 16 were benign angiomyolipomas. Malignant lesions included papillary renal cell carcinoma, chromophobe renal cell carcinoma, and clear-cell renal cell carcinoma, and all were histologically confirmed. Among the benign lesions, 14 angiomyolipomas were classified based on longitudinal ultrasound stability, whereas 2 were confirmed by ultrasound-guided percutaneous biopsy after mild dimensional increase during imaging surveillance.

The mean patient age was 67 years, with a median age of 62 years. Male patients accounted for 65% of the cohort. Malignant lesion diameter ranged from 7 to 28 mm, whereas benign lesion diameter ranged from 9 to 27 mm. All included lesions were visible on conventional B-mode ultrasound, suitable for manual segmentation, and successfully processed for radiomic feature extraction.

The main characteristics of the study cohort and radiomic workflow are summarized in Table 2.

### 3.2. Radiomic Pattern Distribution

**Figure 3:**
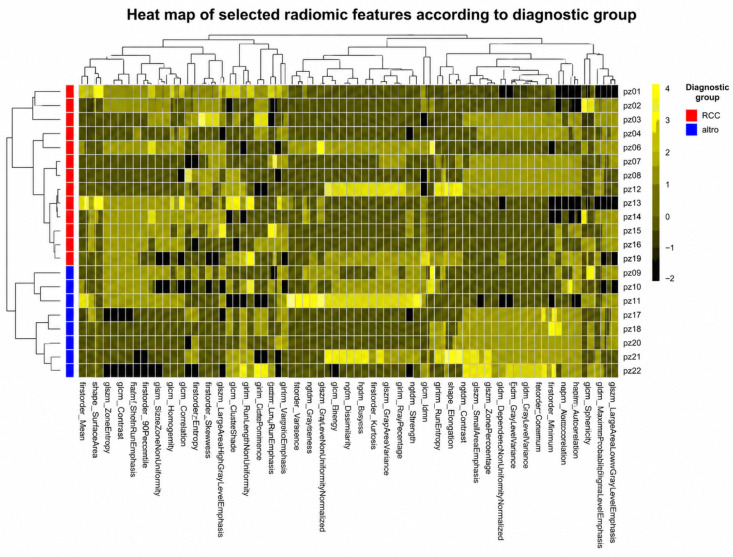
Heat map of all extracted radiomic features across the study cohort. High-resolution unsupervised heat-map visualization of the 114 original radiomic features extracted from manually segmented B-mode ultrasound images of small hyperechoic renal masses. Rows represent individual lesions and columns represent radiomic features. The side annotation indicates the final diagnostic group, including malignant renal cell carcinomas and benign angiomyolipomas. The heat map illustrates global radiomic variability across the cohort and suggests partial clustering tendencies between benign and malignant lesions.

A total of 114 original radiomic features were successfully extracted from each segmented lesion. The extracted features represented complementary quantitative aspects of lesion appearance, including gray-level intensity distribution, two-dimensional shape, and matrix-based texture organization. Unsupervised heat-map visualization of all extracted radiomic features was performed to explore global radiomic variability across lesions and potential clustering tendencies according to the final diagnostic group, defined as benign versus malignant lesions (Figure 3).

**Figure 4:**
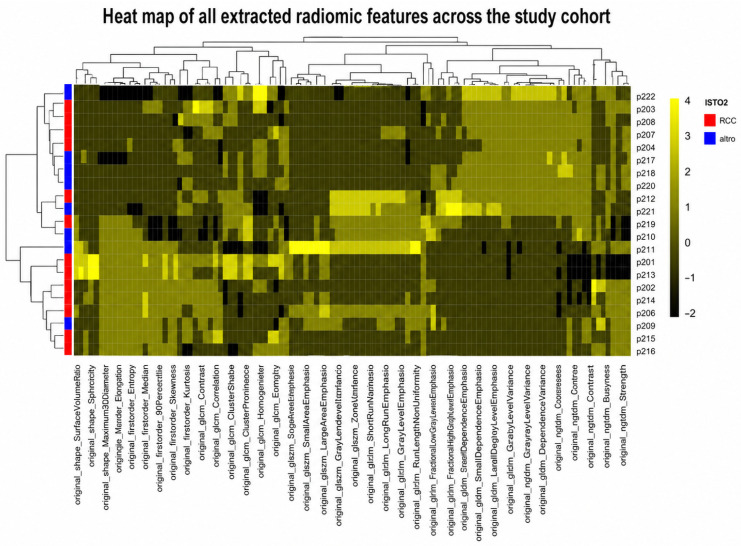
Heat map of selected radiomic features according to diagnostic group. High-resolution heat-map visualization of the radiomic features showing the highest exploratory discriminatory performance for differentiating benign and malignant small hyperechoic renal masses. Rows represent individual lesions and columns represent selected radiomic features. Feature labels were enlarged to improve readability. The side annotation indicates the final diagnostic group, including malignant renal cell carcinomas and benign angiomyolipomas. The selected features show partial clustering tendencies between diagnostic groups, supporting exploratory radiomic separability.

Exploratory heat-map visualization showed partial clustering tendencies between benign and malignant lesions. Although overlap between groups was present, selected radiomic patterns appeared to differ between diagnostic categories. After feature ranking according to discriminatory performance, a selected subset of radiomic features was visualized to assess whether the main separation trend between benign and malignant lesions was preserved (Figure 4).

The selected feature subset preserved part of the separation pattern observed in the global heat map, suggesting that a limited number of radiomic variables may account for a relevant proportion of the quantitative differences between benign and malignant small hyperechoic renal masses. Although overlap between groups remained evident, this exploratory finding supports the hypothesis that specific radiomic features may capture differences between malignant renal cell carcinomas and benign angiomyolipomas.

### 3.3. ROC Analysis

**Figure 5:**
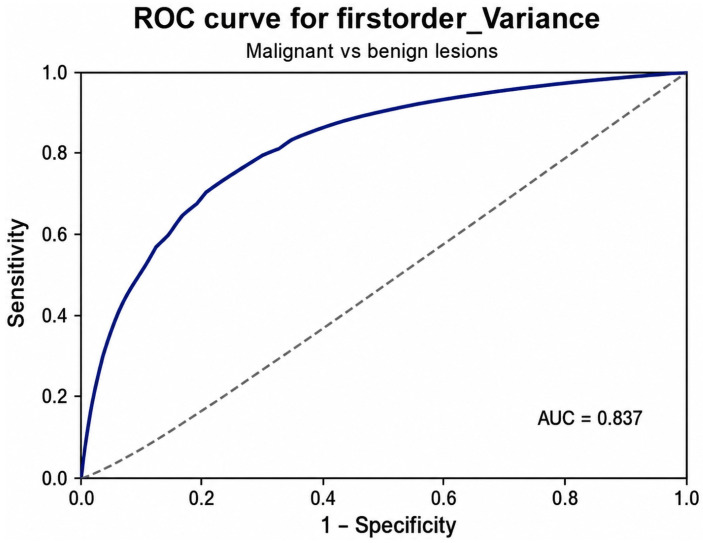
ROC curve for firstorder_Variance in differentiating benign and malignant small hyperechoic renal masses. Receiver operating characteristic curve showing the exploratory discriminatory performance of firstorder_Variance for differentiating malignant renal cell carcinomas from benign angiomyolipomas. The feature achieved an AUC of 0.837 in the exploratory cohort. The dashed diagonal line represents the line of no discrimination, corresponding to random classification performance.

Among the 114 original radiomic features extracted from B-mode ultrasound images, firstorder_Variance showed the highest exploratory discriminatory performance for differentiating benign and malignant small hyperechoic renal masses, yielding an AUC of 0.837. firstorder_MeanAbsoluteDeviation showed a concordant performance, also yielding an AUC of 0.837. After Benjamini–Hochberg false discovery rate correction for the 114 evaluated radiomic features, firstorder_Variance did not retain statistical significance after multiple-comparison adjustment, with an FDR-adjusted q value of 0.70. Therefore, the observed AUC of 0.837 should be interpreted as an exploratory feature-ranking result rather than as confirmatory inferential evidence. firstorder_MeanAbsoluteDeviation showed concordant performance, consistent with its mathematical relationship with firstorder_Variance as a related measure of gray-level dispersion. Overall, these findings support the hypothesis-generating relevance of grayscale dispersion features, but they do not establish a definitive diagnostic biomarker or validated radiomic signature. Both features are first-order measures of gray-level dispersion around the mean intensity and are therefore mathematically related, although not identical. The identical AUC values likely reflect a similar ranking of lesions according to global intralesional grayscale dispersion rather than independent diagnostic information from two unrelated biomarkers. The bootstrap-estimated 95% confidence interval was 0.717–0.957 for both features. Both variables are first-order intensity-based radiomic features that quantify gray-level dispersion within the segmented lesion. Specifically, firstorder_Variance quantifies the mean squared deviation of intensity values from the mean, whereas firstorder_MeanAbsoluteDeviation quantifies the mean absolute deviation from the mean. Therefore, both features describe related aspects of intensity dispersion, with Variance retained as the representative candidate feature for interpretation. The ROC curve for firstorder_Variance is shown in Figure 5.

**Figure 6:**
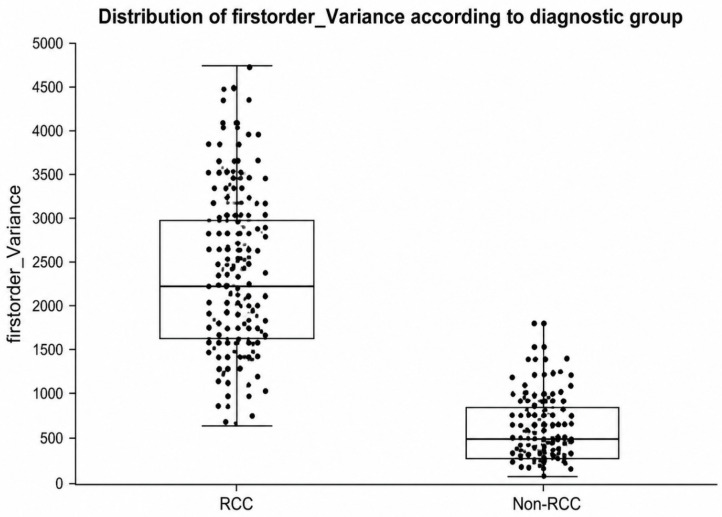
Distribution of firstorder_Variance according to diagnostic group. Boxplot of firstorder_Variance values in malignant renal cell carcinomas and benign angiomyolipomas. Malignant lesions showed higher values than benign lesions, supporting the association between increased gray-level dispersion and malignant diagnosis.

The distribution of firstorder_Variance according to diagnostic group is shown in Figure 6.

The best-performing radiomic features and their diagnostic interpretation are summarized in Table 3.

In this cohort, higher values of both firstorder_Variance and firstorder_MeanAbsoluteDeviation were observed in malignant renal cell carcinomas compared with benign angiomyolipomas, suggesting greater global intralesional grayscale heterogeneity in malignant lesions.

The AUC value of 0.837 indicates good exploratory discrimination in this preliminary cohort. However, because of the retrospective single-center design, limited sample size, multiple feature testing, and absence of external validation, these results should be interpreted as candidate biomarker findings rather than definitive diagnostic performance estimates.

## 4. Discussion

This exploratory single-center study suggests that B-mode ultrasound radiomics may provide complementary quantitative information for differentiating benign and malignant small hyperechoic renal masses. In a final cohort of 42 lesions, including 26 histologically confirmed renal cell carcinomas and 16 benign angiomyolipomas, the representative best-performing feature was firstorder_Variance, which achieved an AUC of 0.837; firstorder_MeanAbsoluteDeviation showed concordant performance but was interpreted as a related measure of grayscale dispersion rather than an independent biomarker. These features are first-order measures of gray-level dispersion and were higher in malignant lesions, suggesting that renal cell carcinomas may show greater global intralesional grayscale heterogeneity than angiomyolipomas on B-mode ultrasound.

The clinical scenario addressed in this study is relevant and frequently encountered in routine radiological practice. Small hyperechoic renal masses are often detected incidentally during conventional abdominal ultrasound examinations [10,44,45]. In many cases, a small, sharply demarcated, hyperechoic renal lesion is presumed to represent an angiomyolipoma. However, hyperechogenicity is not specific for benignity, and renal cell carcinoma may also appear hyperechoic, particularly when small. This overlap creates diagnostic uncertainty at the first-line imaging stage and may influence subsequent patient management, including surveillance, additional imaging, biopsy, ablative treatment, or surgery [46,47,48].

The originality of the present study lies in its focused clinical target. Rather than evaluating renal masses as a broad and heterogeneous category, this work specifically investigates small hyperechoic renal masses measuring ≤3 cm, a subgroup in which conventional B-mode ultrasound is often the first imaging modality but qualitative visual assessment may be insufficient for confident benign versus malignant differentiation. This focused approach is clinically meaningful because the main diagnostic challenge is not lesion detection, but early risk stratification of an apparently benign hyperechoic mass [49,50].

The main finding of this study is that first-order grayscale dispersion features showed the highest exploratory discriminatory performance. Among them, firstorder_Variance was selected as the representative candidate biomarker because of its direct mathematical interpretation as the mean squared dispersion of gray-level values around the lesion mean. firstorder_MeanAbsoluteDeviation showed concordant performance, but it should be interpreted as a related dispersion metric rather than as an independent biomarker.

These findings suggest that quantitative grayscale dispersion features may capture aspects of intralesional heterogeneity that are not consistently appreciable on qualitative B-mode ultrasound assessment alone. Thus, B-mode radiomics may provide an additional quantitative layer to conventional visual interpretation, particularly in lesions with overlapping hyperechoic appearance.

This finding is biologically plausible. Renal cell carcinomas may show variable internal architecture related to heterogeneous cellularity, vascularity, fibrosis, hemorrhagic components, microcystic change, necrosis, or complex tissue composition. These histological characteristics may generate greater variability in B-mode grayscale intensities. Conversely, angiomyolipomas, particularly fat-rich lesions, often show a more homogeneous hyperechoic pattern.

An important aspect of the present results is their interpretability. In radiomics, complex texture features and multivariable models may achieve apparently high performance but are often difficult to explain and may be vulnerable to overfitting, particularly in small datasets. In this study, the most promising features were simple first-order metrics with a clear quantitative meaning. This is advantageous from a translational perspective, because features such as variance and mean absolute deviation are easier to understand, reproduce, and potentially integrate into radiological decision-support workflows than more complex high-dimensional signatures. The present analysis should therefore not be interpreted as the development of a complete radiomic classifier. Rather, it represents an exploratory step aimed at identifying simple, reproducible, and biologically interpretable grayscale descriptors that may be suitable for future validation. In larger multicenter cohorts, these candidate features could be combined with additional radiomic variables, conventional ultrasound descriptors, clinical parameters, and cross-validation strategies to develop and test multivariable machine-learning classifiers. In the present dataset, however, prioritizing interpretability and limiting model complexity was considered more appropriate than training a potentially unstable multifeature classifier.

These findings also support the concept that B-mode ultrasound radiomics should be interpreted as an adjunctive quantitative layer rather than a replacement for expert radiological evaluation.

**Figure 7:**
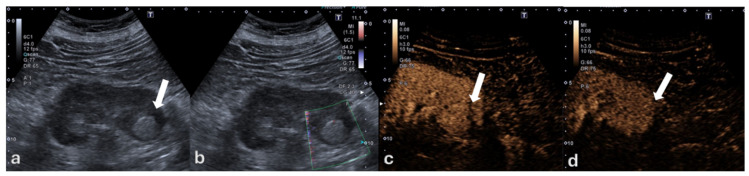
Representative imaging example of a small hyperechoic renal mass with contrast-enhanced ultrasound behavior suspicious for malignancy. Representative case of a small hyperechoic renal mass detected on conventional B-mode ultrasound. (**a**) B-mode image shows a small hyperechoic renal lesion, indicated by the white arrow. (**b**) Color Doppler image shows limited intralesional vascular signal. (**c**) Contrast-enhanced ultrasound image obtained during the early enhancement phase shows enhancement of the lesion relative to the adjacent renal parenchyma. (**d**) Late-phase contrast-enhanced ultrasound image shows relative wash-out of the lesion, a behavior considered suspicious for malignancy. This figure is provided for illustrative purposes only; contrast-enhanced ultrasound findings were not included in radiomic feature extraction or statistical analysis.

**Figure 8:**
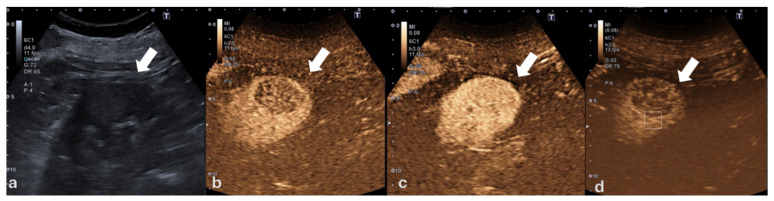
Representative imaging example of a small hyperechoic renal mass with contrast-enhanced ultrasound behavior supporting benignity. B-mode ultrasound shows a small hyperechoic renal lesion, indicated by the white arrow (**a**). Contrast-enhanced ultrasound images obtained during the subsequent vascular phases demonstrate persistent lesion enhancement without relative wash-out compared with the adjacent renal parenchyma (**b**–**d**). This contrast-enhanced behavior was considered supportive of benignity within the clinical diagnostic pathway. The figure is provided for illustrative purposes only; contrast-enhanced ultrasound findings were not included in radiomic feature extraction or statistical analysis.

Conventional ultrasound remains essential for lesion detection, anatomical localization, assessment of margins, echogenicity, acoustic behavior, and relationship with adjacent renal parenchyma. Representative examples are shown in Figure 7 and Figure 8 to illustrate the clinical context in which B-mode radiomics may be applied. Small hyperechoic renal lesions may show overlapping appearances on conventional ultrasound but different downstream imaging behavior during clinical work-up. Importantly, contrast-enhanced ultrasound findings were not included in the present radiomic feature extraction or statistical analysis.

Radiomics may add objective information by quantifying grayscale heterogeneity within the lesion. In this sense, radiomic features could help radiologists express diagnostic uncertainty more quantitatively and support multidisciplinary decision-making in selected equivocal cases.

From a practical perspective, if validated, grayscale dispersion features could contribute to risk stratification of small hyperechoic renal masses. From an implementation perspective, the most realistic initial clinical workflow would be semi-automated rather than fully automated. After acquisition of a standardized B-mode image, the radiologist could select the representative frame and either manually delineate or verify a semi-automatically generated lesion ROI. Predefined radiomic features, such as firstorder_Variance, could then be extracted automatically by dedicated software integrated into the ultrasound workstation, PACS, or an offline decision-support platform. The resulting quantitative value would not provide a standalone diagnosis, but could be displayed as an adjunctive risk-support parameter together with conventional ultrasound findings, lesion size, clinical context, and radiologist confidence.

In the future, fully automated analysis may be feasible if reliable algorithms for lesion detection, boundary delineation, and quality control become available for small renal masses on B-mode ultrasound. However, such implementation would require robust validation across scanners, operators, presets, and institutions. Therefore, at the present stage, the identified radiomic biomarkers should be viewed as candidate quantitative descriptors suitable for future integration into semi-automated ultrasound workflows rather than as immediately deployable automated diagnostic tools.

Lesions showing higher dispersion values may deserve closer attention and further diagnostic evaluation, whereas lesions with low-risk quantitative profiles may be more confidently managed conservatively when supported by clinical context, imaging appearance, and longitudinal stability. This potential application remains preliminary, but it provides a rational basis for future prospective studies.

The conservative statistical approach adopted in this study is appropriate for its exploratory design. No multivariable predictive model was developed because of the limited sample size and the relatively high number of extracted radiomic features. This choice reduces the risk of overfitting and avoids overstating the clinical performance of a model derived from a small single-center cohort. Instead, the analysis focused on feature-level discrimination and biological interpretability. The observed AUC of 0.837 for both firstorder_Variance and firstorder_MeanAbsoluteDeviation should therefore be considered encouraging but preliminary.

The exploratory performance observed in the present study is broadly consistent with previously reported radiomics studies on renal mass characterization, although direct comparisons should be made with caution. In a larger ultrasound-based radiomics study of solid renal masses, multivariable radiomics models and nomograms achieved validation AUC values in the range of approximately 0.86–0.87 for differentiating benign from malignant lesions [51]. CT-based radiomics studies, particularly those focused on differentiating renal cell carcinoma from fat-poor angiomyolipoma, have reported AUC values around 0.90 when machine-learning classifiers and multiphasic CT datasets were used. Similarly, MRI-based radiomics models for differentiating minimal-fat angiomyolipoma from renal cell carcinoma have shown AUC values ranging from approximately 0.88 for single-sequence radiomics to above 0.90 for combined clinical-radiomic models [52].

In this context, the AUC of 0.837 observed for firstorder_Variance in the present cohort appears encouraging, especially considering that our analysis was intentionally restricted to a single interpretable B-mode ultrasound feature rather than a multivariable radiomic signature [53]. However, our results should not be interpreted as directly equivalent to externally validated multiparametric CT or MRI models. The present study differs from prior work because it specifically focused on small hyperechoic renal masses, used conventional B-mode ultrasound images, and adopted a conservative feature-level approach to reduce overfitting in a limited cohort. Therefore, the identified grayscale dispersion feature should be considered a candidate biomarker for future validation rather than a definitive diagnostic model.

Future research should include multicenter enrollment, standardized B-mode ultrasound acquisition protocols, independent external validation, and reproducibility analysis across readers, scanners, and institutions. Further studies should also evaluate whether radiomic grayscale dispersion features provide incremental value over conventional ultrasound assessment and clinical variables. If confirmed, B-mode ultrasound radiomics could become a practical adjunctive tool for the early characterization of small hyperechoic renal masses and for selecting patients who require further diagnostic work-up or structured surveillance.

## 5. Limitations

This study has several limitations. First, it was retrospective and conducted at a single center, which may have introduced selection bias and limits the generalizability of the findings. Second, the final radiomic cohort was limited, including 42 small hyperechoic renal masses. Although this sample size is acceptable for an exploratory feature-level analysis, it is insufficient for definitive diagnostic validation.

Third, all examinations were performed using a single ultrasound platform. This improved internal standardization but limits direct applicability to other ultrasound systems, vendors, transducers, presets, and acquisition protocols. Ultrasound radiomics is particularly sensitive to technical factors, including gain, depth, focus, acoustic window, patient habitus, and vendor-specific post-processing. Therefore, the robustness of the identified features should be tested across different scanners and acquisition settings.

Fourth, segmentation was performed manually by a single experienced radiologist. Manual segmentation allowed careful anatomical control of the region of interest, which is particularly important for small renal lesions, but it remains operator-dependent. Interobserver and intraobserver reproducibility were not assessed and should be included in future studies.

Fifth, the reference standard was not uniform across all lesions. All malignant lesions were histologically confirmed, whereas most benign angiomyolipomas were classified based on longitudinal ultrasound stability, with biopsy performed in two cases after mild dimensional increase during follow-up. This approach reflects real-world clinical practice, but it may introduce verification bias.

Although multiple-comparison control was performed using the Benjamini–Hochberg false discovery rate procedure, the top-ranked feature did not retain statistical significance after correction. This result highlights the risk of false-positive or unstable feature-level findings in a small cohort with a relatively high number of extracted radiomic features. Therefore, the identified grayscale dispersion features should be interpreted as exploratory, hypothesis-generating candidates requiring external validation rather than as definitive diagnostic biomarkers.

## 6. Clinical Implications

B-mode ultrasound radiomics may represent a promising adjunctive tool for the assessment of small hyperechoic renal masses. In routine practice, these lesions are often presumed to be angiomyolipomas when they appear well defined and hyperechoic, but malignant renal cell carcinomas may show overlapping B-mode features. Quantitative radiomic descriptors of grayscale dispersion may help identify lesions with greater intralesional heterogeneity and therefore support early risk stratification.

The potential role of this approach is not to replace histopathology, advanced imaging, or expert radiological interpretation. Rather, B-mode radiomics may provide an additional quantitative layer extracted from the first-line ultrasound examination. If validated, features such as firstorder_Variance and firstorder_MeanAbsoluteDeviation could help radiologists identify lesions that warrant further diagnostic work-up, biopsy, or closer surveillance, while supporting more conservative management in lesions with low-risk quantitative profiles and stable imaging behavior.

Future studies should evaluate whether these radiomic features provide incremental value over conventional ultrasound assessment, lesion size, clinical variables, and radiologist confidence. Multicenter validation, standardized acquisition protocols, and reproducibility analysis are required before integration into clinical decision-making.

## 7. Conclusions

B-mode ultrasound radiomics is feasible for the quantitative assessment of small hyperechoic renal masses measuring ≤3 cm and may provide complementary information for differentiating benign angiomyolipomas from malignant renal cell carcinomas.

In this exploratory single-center cohort, firstorder_Variance and firstorder_MeanAbsoluteDeviation showed the highest discriminatory performance, each achieving an AUC of 0.837. Higher values were observed in malignant lesions, suggesting that renal cell carcinomas may demonstrate greater global intralesional grayscale heterogeneity than angiomyolipomas on B-mode ultrasound.

These findings suggest that first-order grayscale dispersion features may represent hypothesis-generating candidates for future validation; however, they should not be considered confirmed imaging biomarkers because the top-ranked feature did not retain statistical significance after false discovery rate correction.

## Figures and Tables

**Table 1:** Radiomic feature classes. Overview of the radiomic feature classes extracted from manually segmented B-mode ultrasound images. First-order features describe gray-level intensity distribution, shape-based features describe two-dimensional morphology, and matrix-based texture features characterize spatial relationships and patterns of gray-level organization.

Feature Class	Description
First-order statistics	Distribution of gray-level intensity values within the lesion
Shape-based 2D Features	Two-dimensional lesion morphology
GLCM	Spatial relationship of gray-levels
GLRLM	Distribution of consecutive runs of equal gray level
GLSZM	Distribution of connected zones with the same gray level
NGTDM	Difference between a gray level and neighboring gray levels
GLDM	Gray-level dependencies within the lesion

**Table 2:** Study cohort and radiomic workflow. Summary of the final radiomic cohort, diagnostic group distribution, lesion diameter, reference standards, imaging modality, ultrasound platform, image format, segmentation software, radiomic extraction software, and number of extracted radiomic features.

Variable	Value
Study Design	Retrospective, single center
Study period	July 2022–April 2025
Patients initially enrolled	44
Lesions included in radiomic analysis	42
Mean age	67 years
Median age	62
Male Sex	65%
Malignant Lesions	26
Benign lesions	16
Malignant Histology	Papillary RCC, *n* = 14; chromophobe RCC, *n* = 8; clear-cell RCC, *n* = 4
Malignant Lesion Diameter	7–28 mm
Benign diagnosis AML	16
Benign Lesion Diameter	9–27 mm
Benign reference standard	Ultrasound follow-up stability in 14 lesions; ultrasound-guided percutaneous biopsy in 2 lesions
Image Modality for Radiomics	B-mode ultrasound
Image Format	DICOM
Segmentation software	ITK-SNAP v 3.8.0
Radiomic Extraction	Pyradiomics v 3.0
Extracted radiomic features	114 original features

**Table 3:** Representative first-order grayscale dispersion features for differentiating benign and malignant small hyperechoic renal masses. Summary of the first-order grayscale dispersion features showing the highest and concordant exploratory discriminatory performance in ROC analysis. Because firstorder_Variance and firstorder_MeanAbsoluteDeviation are mathematically related measures of gray-level dispersion, they should not be interpreted as independent biomarkers. Both features belong to the first-order class and quantify gray-level dispersion within the segmented lesion. Higher values were associated with malignant renal cell carcinomas compared with benign angiomyolipomas.

Feature	AUC	95& C.I.	Association
Firstorder_Variance	0.837	0.717–0.957	Higher in malignant lesions
Firstorder_MeanAbsoluteDeviation	0.837	0.717–0.795	Higher in malignant lesions

## Data Availability

Data available on demand to the corresponding author due to privacy reasons.
